# Clinical differences between delayed and acute onset postoperative spinal infection

**DOI:** 10.1097/MD.0000000000029366

**Published:** 2022-06-17

**Authors:** Sung-Woo Choi, Jae Chul Lee, Won Seok Lee, Jin Yeong Hwang, Min Jung Baek, Yoon Seo Choi, Hae-Dong Jang, Byung-Joon Shin

**Affiliations:** a Department of Orthopaedic Surgery, Soonchunhyang University College of Medicine, Seoul, Korea; b Department of Obstetrics and Gynecology, Bundang CHA Hospital, Seongnam, Korea; c Early Childhood Education, Ewha Woman's University, Korea; d Department of Orthopaedic Surgery, Soonchunhyang University College of Medicine, Bucheon, Korea.

**Keywords:** antibiotic prophylaxis, delayed infection, postoperative complication, spondylodiscitis, surgical wound infection

## Abstract

Spine surgeons often encounter cases of delayed postoperative spinal infection (PSI). Delayed-onset PSI is a common clinical problem. However, since many studies have investigated acute PSIs, reports of delayed PSI are rare. The purpose of this study was to compare the clinical features, treatment course, and prognosis of delayed PSI with acute PSI.

Ninety-six patients diagnosed with postoperative spinal infection were enrolled in this study. Patients were classified into 2 groups: acute onset (AO) within 90 days (n = 73) and delayed onset (DO) after 90 days (n = 23). The baseline data, clinical manifestations, specific treatments, and treatment outcomes were compared between the 2 groups.

The history of diabetes mellitus (DM) and metallic instrumentation at index surgery were more DO than the AO group. The causative organisms did not differ between the 2 groups. Redness or heat sensation around the surgical wound was more frequent in the AO group (47.9%) than in the DO group (21.7%) (*P* = .02). The mean C-reactive protein levels during infection diagnosis was 8.9 mg/dL in the AO and 4.0 mg/dL in the DO group (*P* = .02). All patients in the DO group had deep-layer infection. In the DO group, revision surgery and additional instrumentation were required, and the duration of parenteral antibiotic use and total antibiotic use was significantly longer than that in the AO group. Screw loosening, disc space collapse, and instability were higher in the DO group (65.2%) than in the AO group (41.1%) (*P* = .04). However, the length of hospital stay did not differ between the groups.

Delayed-onset PSI requires more extensive and longer treatment than acute-onset surgical site infection. Clinicians should try to detect the surgical site infection as early as possible.

## 1 Introduction

Postoperative spinal infections (PSI) are one of the most common complications of spinal surgery.^[1–3]^ Compared to other orthopedic surgical interventions, the incidence of postoperative infections in spine surgery is higher, has more severe complications,^[4]^ and can cause pseudoarthrosis, chronic pain, deformity, neurologic sequelae, and even death. These infections result in longer hospital stays, higher revision surgery rates, and even higher mortality, with the average additional cost for each patient being $4067–$200,000.^[5,6]^

Although it is important to identify the characteristics of PSI, the symptoms of PSI are unclear, and it is difficult to diagnose postoperative infections. Various authors have described and classified infections based on their diagnosis.^[7,8]^ However, there is no absolute standard separating acute-or delayed-onset infections among the various studies, and acute-onset infections are rarely compared directly to delayed-onset infections. Proper management of delayed infections remains controversial. Differences exist in the treatment methods and prognoses between acute-and delayed-onset infections. Acute phase treatments appear to be simpler and can be treated with intravenous antibiotics alone.^[9]^ In delayed infections, the detection of bacteria is delayed, thereby postponing diagnosis. If an early detection of delayed infections is feasible, it can have a large impact on the overall treatment modality and prognosis of infections after spinal surgery.

The purpose of this study was to identify the risk factors for acute and delayed onset PSI and to compare the clinical features and outcomes to determine the most effective treatment for each type of infection.

## 2 Methods

This retrospective cohort study was approved by the Institutional Review Board (No. 201903011). We used an institution-based electronic registry database with discharge diagnoses of “postoperative infection,” “infectious spondylitis,” “surgical site infection,” “postoperative wound infection” from March 2000 to July 2018 in the orthopedic department of Soonchunhyang University, College of Medicine. Each case was reviewed manually.

We excluded patients who had PSI cervical lesions, less than 2 years of follow-up after infection treatment, and surgical site infection caused by tuberculosis, fungus, or parasite infection. The patients’ baseline data included smoking history, comorbidities (e.g., hypertension, diabetes mellitus, end-stage renal disease, and liver cirrhosis), age, sex, body mass index (BMI), type of index operation, and instrumentation usage, which were used to differentiate between the acute onset (AO) and delayed onset (DO) groups. We described PSI based on the criteria for defining and classifying PSIs in the Centers for Disease Control and Prevention National Health Safety Network for both superficial and deep surgical site infections (SSI).^[10]^ All infections were diagnosed based on patient symptoms (e.g., fever, localized swelling or heating, wound dehiscence, back pain) and inflammatory markers (e.g., erythrocyte sedimentation rate [ESR], C-reactive protein [CRP]) and confirmed by an infectious disease physician and attending surgeon. Blood cultures were performed for every patient before antibiotic exposure. Wound cultures were performed after the wounds were accessed. Two fellowship-trained orthopedic spine surgeons categorized infections into 2 groups, superficial and deep layers, based on their depth. We defined AO PSIs as infections that occurred within 90 days after the index operation and DO PSIs as infections that occurred 90 days after the index operation.^[11–13]^ We compared revision surgeries and antibiotic usage to determine differences in specific treatment methods. In this study, exposure to antibiotics was defined as any use of antibiotics within 2 weeks prior to obtaining culture samples in this study.^[14]^ We defined only those procedures that were performed under general anesthesia as revision surgeries and did not include irrigation or simple debridement under local anesthesia. For PSI treatment outcomes, we analyzed radiologic findings, additional anterior surgeries, and extension of fusion to adjacent segments during the treatment course. Poor radiologic findings were defined as disc space collapse and/or pedicle screw pull-out in the final follow-up images compared with immediate postoperative radiographs associated with deterioration of clinical symptoms.^[15]^ Additional anterior surgery and extension of fusion to adjacent segments were defined as cases that gained a damaged anatomic structure by repeated invasive procedures rather than the planned index operation. Additionally, the length of hospital stay and number of deaths were investigated.

All statistical analyses were performed using SPSS version 26.0 for Windows (SPSS Inc., Chicago, IL, USA). The Chi-Squared test and Fisher exact test were used to determine the differences in proportions for each variable, and the independent samples *t*-test or Mann–Whitney *U* test was used to compare continuous variables between groups. Statistical significance was set at *P* < .05 as statistically significant.

## 3 Results

### 3.1 Baseline data

In this study, we retrospectively evaluated 96 patients. The acute infection group comprised 73 patients who were diagnosed with infections within 90 days of their index operation, and 23 patients were classified as having delayed infections. The mean age of patients at surgery was 69.7 years (range 37–94 years) in the AO group and 68.3 years (range 42–89 years) in the DO group. There were 43 men and 30 women with acute infections, and 16 men and 7 women with delayed infections. The number of patients with a history of diabetes was higher in the DO group (47.8%) than in the AO group (20.5%) (*P* = .01). Metallic instrumentation was used in the index operations in 40 patients (54.8%) in the AO group and 18 patients (78.3%) in the DO group (*P* = .04). There were no significant differences in age, sex, BMI, type of index operation, or postoperative albumin levels between the AO and DO groups (Table 1).

**Table 1:** Baseline data between acute onset (AO) and delayed onset (DO) patients.

Variable	Acute (n = 73)	Delayed (n = 23)	*P* value
Age, mean (SD)^†^	69.7 ± 12.6	68.3 ± 11.8	.468
Sex			.360
Male	43 (58.6%)	16 (69.6%)	
Female	30 (41.1%)	7 (30.4%)	
Comorbidity			
Cigarette smoker	30 (41.1%)	9 (39.1%)	.867
Hypertension	37 (50.7%)	11 (47.8%)	.811
Diabetes mellitus	15 (20.5%)	11 (47.8%)	.010^∗^
Liver cirrhosis^¥^	3 (4.1%)	0 (0.0%)	1.000
Hemodialysis^¥^	2 (2.7%)	0 (0.0%)	1.000
Old age (>70)	40 (54.8%)	12 (52.2%)	.826
No. of co-morbidity^†^			.273
No. <3	56 (76.7%)	15 (65.2%)	
No. ≥3	17 (23.3%)	8 (34.8%)	
BMI, mean (SD)^†^	24.2 ± 3.0	24.1 ± 3.8	.925
Index operation^¥^			.629
Posterior fusion	40 (54.8%)	16 (69.6%)	
Anterior fusion	4 (5.5%)	2 (8.7%)	
Anterior and posterior fusion	22 (30.1%)	1 (4.3%)	
Decompression	23 (31.6%)	4 (17.4%)	
Metallic instrumentation	40 (54.8%)	18 (78.3%)	.045^∗^
Operation time, mean (SD)^†^	179.21 ± 116.8	128.4 ± 81.9	.063
Postop. albumin level (mg/dL), mean (SD)^†^	3.1 ± 0.5	3.1 ± 0.7	.914

No = number, Postop = postoperative.

∗ *P* value <.05.

¥ *P* value by fisher's exact test.

‡ *P* value by Chi-Squared test.

† *P* value by Mann–Whitney *U* test.

### 3.2 Clinical characteristics

Among the 5 SSI symptoms/signs (back pain, localized swelling, redness or heating sensation, wound dehiscence, and fever [≥38°C]), redness or heating sensation was more frequent in the AO group (47.9%) than in the DO group (21.7%) (*P* = .02). There were no significant differences in the culture results.

ESR levels were similar between the 2 groups. The mean CRP level was higher in the AO group (8.9 mg/dL) than in the DO group (4.0 mg/dL) (*P* = .02). Antibiotic exposure and duration were similar between the 2 groups. All patients in the DO group had deep SSIs, but only 74% of those in the AO group had deep SSIs (*P* = .005) (Table 2).

**Table 2:** Clinical characteristics between acute onset (AO) and delayed onset (DO) patients.

Variable	Acute (n = 73)	Delayed (n = 23)	*P* value^‡^
Culture results			
MSSA^¥^	4 (5.5%)	1 (4.3%)	1.000
MRSA^¥^	9 (12.3%)	6 (26.1%)	.184
*Staphylococcus epidermidis*	19 (26.0%)	7 (30.4%)	.678
*Enterococcus faecalis*^¥^	4 (5.5%)	1 (4.3%)	1.000
*Escherichia coli*^¥^	2 (2.7%)	0 (0.0%)	1.000
*Enterobacter clocae*^¥^	0 (0.0%)	1 (4.3%)	.240
*Pseudomonas aeruginosa*^¥^	0 (0.0%)	2 (8.7%)	.055
Culture negative yield	27 (37.0%)	12 (52.2%)	.196
Symptoms/signs			
Increasing or persisting back pain ^¥^	62 (84.9%)	22 (95.7%)	.283
Localized swelling ^¥^	18 (24.7%)	2 (8.7%)	.142
Redness or feeling of heat	35 (47.9%)	5 (21.7%)	.026^∗^
Wound dehiscence ^¥^	8 (11.0%)	2 (8.7%)	1.000
Fever (>38°C)	20 (27.4%)	3 (13.0%)	.160
ESR level (mm/h), mean (SD)^†^	81.3 ± 29.6	88.4 ± 29.2	.400
CRP level (mg/dL), mean (SD)^†^	8.9 ± 12.4	4.0 ± 3.1	.020^∗^
Antibiotics exposure	41 (56.2%)	10 (43.5%)	.288
Exposure duration (days), mean (SD)^†^	4.5 ± 6.6	6.7 ± 15.5	.618
Layer of infection^¥^			.005^∗^
Superficial	19 (26.0%)	0 (0.0%)	
Deep	54 (74.0%)	23 (100.0%)	

MSSA = Methicillin-sensitive *Staphylococcus aureus*, MRSA = Methicillin-resistant *Staphylococcus aureus*, ESR = erythrocyte sedimentation rate, CRP = C-reactive protein.

∗ *P* value <.05.

¥ *P* value by fisher's exact test.

‡ *P* value by Chi-Squared test.

† *P* value by Mann–Whitney *U* test.

### 3.3 Specific PSI treatments and results

Revision surgery was performed in all patients in the DO group (23 patients) and 79.5% (58 patients) in the AO group (*P* = .01). The revision methods varied. Incision and drainage were performed in 38.4% of the AO group (28 cases) and in 13% of the DO group (3 cases). Instrumentation use was more frequent in the DO group (13 cases, 56.5%) than in the AO group (22 cases, 30.1%). There were no significant differences in the number of revisions between the 2 groups. The duration of antibiotic use was significantly different between the 2 groups. The mean duration of parenteral antibiotic usage was 38.1 days in the AO group and 45.0 days in the DO group (*P* = .03). The total antibiotic usage (intravenous + oral) duration was longer in the DO group (68.5 days) than in the AO group (51.7 days) (*P* = .01) (Table 3).

**Table 3:** Specific treatment between acute onset (AO) and delayed onset (DO) patients.

Variable	Acute (n = 73)	Delayed (n = 23)	*P* value^‡^
Revision surgery	58 (79.5%)	23 (100%)	.018^∗^
Revision method^¥^			.001^∗^
I & D	28 (38.4%)	3 (13.0%)	
Decompression	2 (2.7%)	1 (4.3%)	
Instrumentation	22 (30.1%)	13 (56.5%)	
Removal of implant	6 (8.2%)	6 (26.1%)	
No. of revision surgery, mean (SD)^†^	1.1 ± 0.8	1.3 ± 0.5	.137
Duration (days) of parenteral antibiotics treatment, mean (SD)^†^	38.1 ± 21.6	45.0 ± 17.1	.039^∗^
Duration (days) of oral antibiotics treatment, mean (SD)^†^	13.9 ± 12.5	21.1 ± 21.5	.094
Duration (days) of total antibiotics treatment, mean (SD)^†^	51.7 ± 27.6	68.5 ± 38.0	.012^∗^

I&D = incision and drainage.

¥ *P* value by fisher's exact test.

‡ *P* value by Chi-Squared test.

† *P* value by Mann–Whitney *U* test.

Poor radiologic findings were more common in the DO group (65.2%) than in the AO group (41.1%) (*P* = .04). However, the rates of performing additional anterior surgeries and extensions of fusion to adjacent segments were similar between the 2 groups. The length of hospital stays was 74.2 days in the AO group and 64.7 days in the DO group, which was not statistically different (Table 4). Only 1 patient in the AO group died during the treatment course.

**Table 4:** Final results between acute onset (AO) and delayed onset (DO) patients.

Variable	Acute (n = 73)	Delayed (n = 23)	*P* value^‡^
Poor radiologic findings (loosening, collapse, instability)	30 (41.1%)	15 (65.2%)	.043^∗^
Anterior surgical approach	20 (27.4%)	10 (43.5%)	.147
Additional segment surgery	9 (12.3%)	6 (26.1%)	.184
Length of hospital stay, mean (SD)^†^	74.2 ± 39.2	64.7 ± 39.2	.172
Death^¥^	1 (4.4%)	0 (0%)	N/A

‡ *P* value by Chi-Squared test.

† *P* value by Mann–Whitney *U* test.

¥ *P* value by fisher's exact test. N/A = not available.

## 4 Discussion

Among the 96 patients who met our PSI criteria, 24.0% of all infections occurred in the DO group. The rates of delayed infection have been reported to be 4.3%^[16]^ and 16.7%^[7]^ in previous studies. Compared to these studies, our delayed infection prevalence was much higher, which might be attributable to our definition of delayed infection. Some studies define delayed infection as occurring 9 months after the index operation,^[17–19]^ while others claim that a time period of only 4 weeks or 6 months constitutes delayed infection.^[7,8]^ The definition of the duration of delayed infection remains controversial. For accurate analysis of delayed infections, it is important to establish a precise definition of the duration of postoperative infections.

Acute-and delayed-onset infections are known to have different pathological mechanisms. Viola et al explained that acute-onset infections are derived from a slow wound healing process. However, delayed onset infections are associated with implants.^[20]^ Implants can incubate bacteria and cause delayed infections.^[21]^ In our study, patients who had implants inserted during their index operation had a greater chance of developing delayed infection. This result is consistent with those of previous studies. In patients with implants, bacteria can attach to the structure, form multiple colonies, and develop a glycocalyx membrane, resulting in a biofilm that resists host defenses and antibiotics.^[22–24]^ However, after the use of antibiotics was terminated, the bacteria in the biofilm were expressed, and infection could be detected.

Richard et al hypothesized that delayed infection occurs in 2 ways.^[11,25]^ The first is hematogenous seeding and the second is intraoperative seeding. The causative organisms of both pathways have been reported to be *Propionibacterium acnes* and *Staphylococcus epidermidis.*^[25]^ Because these bacteria have low virulence and grow slowly, their detection can be delayed.^[25]^ The slow growth rate and low virulence of *P. acnes* indicate that cultures should be taken for longer time periods, with some studies even suggesting up to 14 days.^[26,27]^ In our study, there was no infection caused by *P. acnes*, and only 19 patients in the AO group and seven patients in the DO group had *S. epidermidis* infections, with no significant differences between the 2 groups. Generally, in our hospital, bacterial wound cultures are routinely examined after 24 and 48 hours of incubation. When colonies were not detected after 48 hours, the media was held, unless a clinician's special order suspected aerobes or slow-growing bacteria.^[28]^ This may be the reason for the lack of P. acnes detection.

Therefore, it is important to identify infectious organisms through appropriate screening for proper treatment. However, low-grade infections caused by less virulent skin bacteria are often neglected because the symptoms are often unclear and can present simply as persistent pain. Recently, several tests have been developed to address this problem. Corvec et al claimed that aerobic and anaerobic cultures should be carried out in different culture media for 5 to 7 days under aerobic conditions and for at least 2 weeks under anaerobic conditions.^[29,30]^ In addition, 16S rRNA polymerase chain reaction (PCR) amplification can be used to confirm the identity of causative organisms, even if the bacteria are unculturable.^[31]^ Although commercial multiplex PCR kits can be used, these test kits cannot identify all organisms, although in some studies, the causative organisms were more frequently identified by multiplex PCRs than by tissue cultures.^[30,32]^ Therefore, it is prudent to consider using this multifaceted approach to achieve an early diagnosis of the causative organisms that can cause delayed infections.

Accurate diagnosis of infections is crucial to determine correct treatment strategies. Physical examination or the recognition of patient symptoms can help in the early diagnosis of infections. According to our study, 35 patients in the AO group complained of heat or color changes at their operative sites, with their mean CRP levels increasing to 8.9 mg/dL. However, 95.7% of the patients in the DO group complained that back pain was their main symptom, not redness or a heating sensation. These results indicate that the diagnosis of delayed-onset infections can be difficult (). Therefore, clinicians should always be aware of the possibility of delayed infections after spine surgeries because clinical features and laboratory test results are not always definitive.

**Figure 1:**
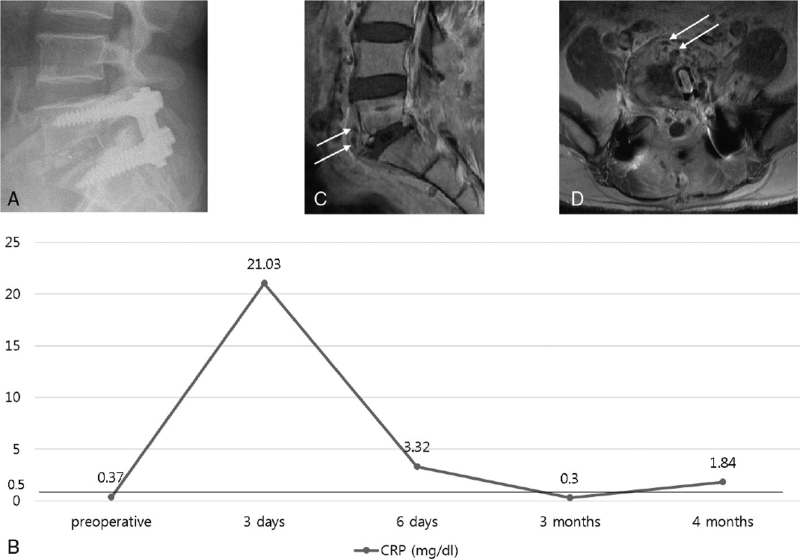
Plain radiographs show no abnormal findings, such as loosening, instability, disc space collapse 4 months after surgery. However, this patient complained un-explained persisting back pain (A). The CRP level reduced to normal range 3 months after surgery, but was slightly increased at an outpatient follow-up 4 months after surgery (B). Contrast-enhanced MRI shows thickening of anterior longitudinal ligament and small abscess pocket at L5-S1 level (white arrow). This is a suspicious sign of postoperative surgical site infection at 4 months after surgery (C, D).

In our study, there were several significant differences in the treatment of infections between the AO and DO groups. First, the incidence of deep-layer infections was higher in the DO group than in the AO group. All patients with delayed infections underwent revision surgeries, while 20.5% of cases in the AO group were treated only with antibiotics. There were also differences in the types of revision surgery between the AO and DO groups. In the AO group, incision and drainage were the most common types of revision surgeries. However, in the DO group, both additional instrumentation and implant removal were required to manage PSIs. Statistically, there was no significant difference in the number of revision surgeries among the patients. However, clinically, the mean number of revision surgeries was higher in the DO group than in the AO group. The duration of antibiotic use also differed between acute and delayed infections. The mean total number of days of antibiotic use was 68.5 days in the DO group and 51.7 days in the AO group. These results indicate that the infection depth was more severe, and the treatment was more difficult in the DO group. As the diagnosis of infection was delayed, more serious and prolonged interventions (antibiotic use or more extensive revision surgeries) were needed for treatment. Additionally, similar to most studies describing the proper management of delayed infections, poor radiologic findings were more frequent in the DO group.^[4,33]^ Consequently, to prevent additional extensive revision surgeries and obtain a better prognosis for patients with PSIs, it is important to identify early signs of infection and prevent delays in proper treatment.

This study had several limitations. First, the delayed onset is not clearly defined. Thus, many previous reports have presented different incidences of delayed-onset infection. Therefore, a direct comparison with other studies is insignificant. In Table 5, the most frequent period was 4 weeks after the index surgery as delayed infection. We chose this period for our study. We believe that 12 weeks is appropriate for this concept. A period of 4 weeks is possible for delayed diagnosis by a clinician, not delayed onset PSI. Second, due to its retrospective design, we were unable to identify the types of bacteria or other microorganisms and in turn their incubation periods. PCR, molecular sonication techniques for implants, and other techniques can be used to detect infectious organisms. However, these methods are not commonly used in clinical practice. For the reason mentioned above, when the organism was not incubated, wound culture lasted just 2 days and blood culture lasted 5 days. Therefore, slow-growing and fussy organisms, such as *P. acnes*, were not detected in this study. However, this was carried out following a clinical laboratory textbook^[28]^ and we think this may reflect a real clinical situation.

**Table 5:** Reported criteria for separate delayed onset infection from acute onset infection in the literature.

Defined duration	Author
≥4 wks	Chaudhary et al,^[34]^ Christodoulou et al,^[35]^ Abhijit et al,^[33]^ Sharif et al,^[36]^ Parchi et al ^[37]^
≥12 wks	Wang et al,^[11]^ Chen et al,^[12]^ Cahill et al,^[13]^ Schömig et al,^[38]^ Hedequist et.al.^[39]^
≥6 mo	Peter et al,^[16]^ Jianxiong et al^[7]^
≥12 mo	Clark et al,^[17]^ Swarup et al ^[40]^

## 5 Conclusion

Delayed-onset PSI mostly involves a deep layer, and internal structures are affected by the organism for a long time. Thus, the treatment method may be more aggressive than that for acute PSI. In cases of delayed-onset PSI, revision surgery was necessary. Moreover, the duration of antibiotic use was longer in patients with delayed onset. From the viewpoint of treatment, delayed-onset PSI results may indicate a worse prognosis than acute-onset PSI. Therefore, early diagnosis is important. However, the symptoms and abnormal laboratory results are not specific to delayed-onset PSI. In the present study, almost all patients with delayed-onset PSI had symptoms of increasing or persisting back pain for a long time after surgery. When a patient has an abnormal pattern of back pain after spinal surgery, the clinician must consider the possibility of delayed SSI, and the diagnostic approach should be a combination of clinical, laboratory, and imaging studies. When a surgeon performs a surgery, highest sensitivity in the diagnosis of PSI must be achieved for which, at least 3 intraoperative tissue samples should be submitted for culture. The removed implant should be sonicated to get a tissue sample to be incubated for a longer period as compared to ordinary culture schedule to detect slow-growing organisms. Although this is a single-center, small patient, and retrospective observational study, we believe it provides a crucial reference for the treatment of delayed-onset PSI.

## Author contributions

**Conceptualization:** Sung-Woo Choi

**Data curation:** Jin Hwang, Jin Yeong Hwang, Yoonseo Choi, Yoon Seo Choi

**Formal analysis:** Min Baek, Min Jung Baek

**Investigation:** Jin Hwang, Jin Yeong Hwang, Min Baek, Min Jung Baek, Won Seok Lee, Yoonseo Choi, Yoon Seo Choi

**Supervision:** Jae Lee, Jae Chul Lee, Sung-Woo Choi

**Validation:** Jae Lee, Jae Chul Lee

**Visualization:** Won Seok Lee

**Writing – original draft:** Jin Yeong Hwang, Jin Hwang, Sung-Woo Choi

**Writing – review & editing:** Jae Lee, Jae Chul Lee, Min Baek, Min Jung Baek, Yoonseo Choi, Yoon Seo Choi

## References

[1] Sharfman ZT Gelfand Y Shah P. Spinal epidural abscess: a review of presentation, management, and medicolegal implications. Asian Spine J 2020;14:742–59. 32718133 10.31616/asj.2019.0369 PMC7595828

[2] Oikonomidis S Altenrath L Westermann L Bredow J Eysel P Scheyerer MJ. Implant-associated infection of long-segment spinal instrumentation: a retrospective analysis of 46 consecutive patients. Asian Spine J 2021;15:234–43. 32703924 10.31616/asj.2019.0391 PMC8055457

[3] Mohamad G Amritanand R David KS Krishnan V Arockiaraj J. Treatment strategy and outcomes in patients with hematogenous culture-negative pyogenic vertebral osteomyelitis. Asian Spine J 2019;13:61–7. 30326685 10.31616/asj.2018.0093 PMC6365777

[4] Lazennec J-Y Fourniols E Lenoir T. Infections in the operated spine: update on risk management and therapeutic strategies. Orthop Traumatol 2011;97:S107–16. 10.1016/j.otsr.2011.07.002 21856262

[5] Thalgott JS Cotler HB Sasso RC LaROCCA H Gardner V. Postoperative infections in spinal implants. Classification and analysis--a multicenter study. Spine 1991;16:981–4. 1948385 10.1097/00007632-199108000-00020

[6] Chahoud J Kanafani Z Kanj SS. Surgical site infections following spine surgery: eliminating the controversies in the diagnosis. Front Med 2014;1:07. 10.3389/fmed.2014.00007 PMC4335387 25705620

[7] Shen J Liang J Yu H Qiu G Xue X Li Z. Risk factors for delayed infections after spinal fusion and instrumentation in patients with scoliosis. J Neurosurg 2014;21:648–52. 10.3171/2014.6.SPINE13636 24995494

[8] Bassewitz H Fischgrund J Herkowitz H. WB SAUNDERS COMPANY. Postoperative spine infections. Seminars Spine Surg 2000;12:203–11.

[9] Kowalski TJ Berbari EF Huddleston PM Steckelberg JM Mandrekar JN Osmon DR. The management and outcome of spinal implant infections: contemporary retrospective cohort study. Clin Infectious Dis 2007;44:913–20. 10.1086/512194 17342641

[10] Mangram AJ Horan TC Pearson ML Silver LC Jarvis WR. Committee HICPA. Guideline for prevention of surgical site infection. Am J Infection Control 1999;27:97–134. 10196487

[11] Wang M Xu L Yang B. Incidence, management and outcome of delayed deep surgical site infection following spinal deformity surgery: 20-Year experience at a single institution. Global Spine J 2020;2192568220978225. 10.1177/2192568220978225 PMC9210238 33375859

[12] Chen SH Lee CH Huang KC Hsieh PH Tsai SY. Postoperative wound infection after posterior spinal instrumentation: analysis of long-term treatment outcomes. Eur Spine J 2015;24:561–70. 25351841 10.1007/s00586-014-3636-9

[13] Cahill PJ Warnick DE Lee MJ. Infection after spinal fusion for pediatric spinal deformity: thirty years of experience at a single institution. Spine 2010;35:1211–7. 20445480 10.1097/BRS.0b013e3181c212d1

[14] Parvizi J Erkocak OF Della Valle CJ. Culture-negative periprosthetic joint infection. J Bone Joint Surg Am 2014;96:430–6. 24599206 10.2106/JBJS.L.01793

[15] Lee JC Baek MJ Choi S-W. Retrospective analysis of culture-negative versus culture-positive postoperative spinal infections. Medicine 2018;97:e10643. 29768329 10.1097/MD.0000000000010643 PMC5976297

[16] Lewkonia P DiPaola C Street J. Incidence and risk of delayed surgical site infection following instrumented lumbar spine fusion. J Clin Neurosci 2016;23:76–80. 26358200 10.1016/j.jocn.2015.05.039

[17] Clark CE Shufflebarger HL. Late-developing infection in instrumented idiopathic scoliosis. Spine 1999;24:1909. 10515015 10.1097/00007632-199909150-00008

[18] Heggeness MH Esses SI Errico T Yuan HA. Late infection of spinal instrumentation by hematogenous seeding. Spine 1993;18:492–6. 8470011

[19] Richards BS. Delayed infections following posterior spinal instrumentation for the treatment of idiopathic scoliosis. JBJS 1995;77:524–9. 10.2106/00004623-199504000-00004 7713968

[20] Viola RW King HA Adler SM Wilson CB. Delayed infection after elective spinal instrumentation and fusion. A retrospective analysis of eight cases. Spine 1997;22:2444–50. discussion 50-1. 9355228 10.1097/00007632-199710150-00023

[21] Shiono Y Ishii K Nagai S. Delayed Propionibacterium acnes surgical site infections occur only in the presence of an implant. Scientific Rep 2016;6:01–10. 10.1038/srep32758 PMC5018724 27615686

[22] Sampedro MF Huddleston PM Piper KE. A biofilm approach to detect bacteria on removed spinal implants. Spine 2010;35:1218–24. 20445479 10.1097/BRS.0b013e3181c3b2f3

[23] McConoughey SJ Howlin R Granger JF. Biofilms in periprosthetic orthopedic infections. Future Microbiol 2014;9:987–1007. 25302955 10.2217/fmb.14.64 PMC4407677

[24] Kapadia BH Berg RA Daley JA Fritz J Bhave A Mont MA. Periprosthetic joint infection. Lancet 2016;387:386–94. 26135702 10.1016/S0140-6736(14)61798-0

[25] Richards BS Emara KM. Delayed infections after posterior TSRH spinal instrumentation for idiopathic scoliosis: revisited. Spine 2001;26:1990–5. 11547197 10.1097/00007632-200109150-00009

[26] Zeller V Ghorbani A Strady C Leonard P Mamoudy P Desplaces N. Propionibacterium acnes: an agent of prosthetic joint infection and colonization. J Infection 2007;55:119–24. 10.1016/j.jinf.2007.02.006 17418419

[27] Corvec S Portillo ME Pasticci BM Borens O Trampuz A. Epidemiology and new developments in the diagnosis of prosthetic joint infection. Int J Artificial Organs 2012;35:923–34. 10.5301/ijao.5000168 23138706

[28] McPherson RA. Henry's Clinical Diagnosis and Management by Laboratory Methods: First South Asia Edition_e-Book: Elsevier India 2017;1:606.

[29] Aubin G Portillo M Trampuz A Corvec S. Propionibacterium acnes, an emerging pathogen: from acne to implant-infections, from phylotype to resistance. Med Maladies Infectieuses 2014;44:241–50. 10.1016/j.medmal.2014.02.004 24656842

[30] Portillo ME Corvec S Borens O Trampuz A. Propionibacterium acnes: an underestimated pathogen in implant-associated infections. BioMed Res Int 2013;2013:10. 10.1155/2013/804391 PMC3838805 24308006

[31] Clarridge JE. Impact of 16S rRNA gene sequence analysis for identification of bacteria on clinical microbiology and infectious diseases. Clin Microbiol Rev 2004;17:840–62. 15489351 10.1128/CMR.17.4.840-862.2004 PMC523561

[32] Achermann Y Vogt M Leunig M Wüst J Trampuz A. Improved diagnosis of periprosthetic joint infection by multiplex PCR of sonication fluid from removed implants. J Clin Microbiol 2010;48:1208–14. 20164283 10.1128/JCM.00006-10 PMC2849575

[33] Pawar AY Biswas SK. Postoperative spine infections. Asian Spine J 2016;10:176–83. 26949475 10.4184/asj.2016.10.1.176 PMC4764532

[34] Chaudhary SB Vives MJ Basra SK Reiter MF. Postoperative Spinal Wound Infections and Postprocedural Diskitis. The Journal of Spinal Cord Medicine 2007;30:441–51. 18092559 10.1080/10790268.2007.11753476 PMC2141723

[35] Christodoulou AG Givissis P Symeonidis PD Karataglis D Pournaras J. Reduction of postoperative spinal infections based on an etiologic protocol. Clin Orthop Related Res 2006;444:107–13. 10.1097/01.blo.0000201174.10506.cc 16523134

[36] Sharif S Gulzar F. postoperative infections of the spine. World Spinal Column J 2015;6:19–26.

[37] Parchi PD Evangelisti G Andreani L. Postoperative spine infections. Orthop Rev (Pavia) 2015;7:5900. 26605028 10.4081/or.2015.5900 PMC4592931

[38] Schomig F Putzier M. Clinical presentation and diagnosis of delayed postoperative spinal implant infection. J Spine Surg 2020;6:772–6. 33447682 10.21037/jss-20-499 PMC7797807

[39] Hedequist D Haugen A Hresko T Emans J. Failure of attempted implant retention in spinal deformity delayed surgical site infections. Spine 2009;34:60–4. 19077923 10.1097/BRS.0b013e31818ed75e

[40] Swarup I Gruskay J Price M. Propionibacterium acnes infections in patients with idiopathic scoliosis: a case-control study and review of the literature. J Child Orthop 2018;12:173–80. 29707057 10.1302/1863-2548.12.170212 PMC5902752

